# Structural Characterization of Daunomycin-Peptide Conjugates by Various Tandem Mass Spectrometric Techniques

**DOI:** 10.3390/ijms22041648

**Published:** 2021-02-06

**Authors:** Adina Borbély, Lilla Pethő, Ildikó Szabó, Mohammed Al-Majidi, Arnold Steckel, Tibor Nagy, Sándor Kéki, Gergő Kalló, Éva Csősz, Gábor Mező, Gitta Schlosser

**Affiliations:** 1 MTA-ELTE Lendület Ion Mobility Mass Spectrometry Research Group and Department of Analytical Chemistry, ELTE Eötvös Loránd University, Pázmány Péter sétány 1/A, H-1117 Budapest, Hungary; adina.borbely@outlook.com (A.B.); mohideenmoscow@gmail.com (M.A.-M.); starnold@caesar.elte.hu (A.S.); 2 Eötvös Loránd Research Network, Supported Research Groups, Research Group of Peptide Chemistry, Pázmány Péter sétány 1/A, H-1117 Budapest, Hungary; lilla.petho@chem.elte.hu (L.P.); szaboi8@gmail.com (I.S.); gmezo@caesar.elte.hu (G.M.); 3 Hevesy György PhD School of Chemistry, ELTE Eötvös Loránd University, Pázmány Péter sétány 1/A, H-1117 Budapest, Hungary; 4 Department of Applied Chemistry, Faculty of Science and Technology, University of Debrecen, Egyetem tér 1, H-4032 Debrecen, Hungary; nagy.tibor@science.unideb.hu (T.N.); keki.sandor@science.unideb.hu (S.K.); 5 Proteomics Core Facility, Department of Biochemistry and Molecular Biology, Faculty of Medicine, University of Debrecen, Egyetem tér 1, H-4032 Debrecen, Hungary; kallo.gergo@med.unideb.hu (G.K.); cseva@med.unideb.hu (É.C.); 6 Department of Organic Chemistry, ELTE Eötvös Loránd University, Pázmány Péter sétány 1/A, H-1117 Budapest, Hungary

**Keywords:** anthracyclines, bioconjugates, collision-induced dissociation, electron predator, high resolution mass spectrometry, MALDI

## Abstract

The use of peptide-drug conjugates has generated wide interest as targeted antitumor therapeutics. The anthracycline antibiotic, daunomycin, is a widely used anticancer agent and it is often conjugated to different tumor homing peptides. However, comprehensive analytical characterization of these conjugates via tandem mass spectrometry (MS/MS) is challenging due to the lability of the O-glycosidic bond and the appearance of MS/MS fragment ions with little structural information. Therefore, we aimed to investigate the optimal fragmentation conditions that suppress the prevalent dissociation of the anthracycline drug and provide good sequence coverage. In this study, we comprehensively compared the performance of common fragmentation techniques, such as higher energy collisional dissociation (HCD), electron transfer dissociation (ETD), electron-transfer higher energy collisional dissociation (EThcD) and matrix-assisted laser desorption/ionization–tandem time-of-flight (MALDI-TOF/TOF) activation methods for the structural identification of synthetic daunomycin-peptide conjugates by high-resolution tandem mass spectrometry. Our results showed that peptide backbone fragmentation was inhibited by applying electron-based dissociation methods to conjugates, most possibly due to the “electron predator” effect of the daunomycin. We found that efficient HCD fragmentation was largely influenced by several factors, such as amino acid sequences, charge states and HCD energy. High energy HCD and MALDI-TOF/TOF combined with collision induced dissociation (CID) mode are the methods of choice to unambiguously assign the sequence, localize different conjugation sites and differentiate conjugate isomers.

## 1. Introduction

In recent years, peptide-drug conjugates (PDCs) have become an effective approach for the targeted delivery of therapeutic agents [1]. In this strategy one or more chemotherapeutic agents are covalently attached to peptides which can serve as carriers to selectively deliver cytotoxic drugs to solid malignancies. PDCs aim to overcome the limitations of traditional chemotherapy and ensure high therapeutic efficacy with low toxic side effects, thereby improving the quality of life of patients receiving anticancer treatment. Hence, peptide-targeted drugs are promising tools for modern precision medicine [1,2,3].

The α-melanocyte-stimulating hormone (α-MSH, *Ac*-SYSMEHFRWGKPV-*NH_2_*), the adrenocorticotropic hormone (ACTH, SYSMEHFRWGKPVGKKRRPVKVYPNGAEDESAEAFPLEF), and other related peptides are produced by the proteolytic cleavage of the polypeptide precursor proopiomelanocortin (POMC) [4]. Given that α-MSH peptide analogs specifically bind to melanocortin-1 (MC1) receptors, they are promising drug carriers in melanoma therapy [5]. The analog containing Nle at position 4, as well as the central fragments containing lysine residues exhibit high melanotropic activity, thus providing a wide platform for PDC development with different sites for drug conjugation [6].

Over the last few years, our research group has intensively focused on the development of PDCs based on the anthracycline antitumor antibiotic, daunomycin (Dau). Daunomycin has been conjugated to several cancer-specific peptides and the biological properties of the resulting bioconjugates have been investigated in detail [7,8,9]. Several promising new compounds that exert strong in vivo antitumor effect are in preclinical development [10,11,12]. The comprehensive analytical characterization of drug candidates is not only a key step at various stages of drug development, but also a fundamental requirement of the regulatory authorities [13]. Tandem mass spectrometry (MS/MS) is an effective technique for providing extensive data that supports the structural identification of PDCs. 

Significant spontaneous fragmentation of anthracycline containing conjugates during electrospray ionization (ESI) has a negative effect on the quality and reliability of the resulting ESI-MS data, thereby hindering the conduct of mass spectrometric analysis of these compounds [14,15]. Daunomycin consists of a tetracyclic quinoid aglycone and a daunosamine sugar moiety attached to the aglycon part through an *O*-glycosidic bond. Mass spectrometric analysis by ESI indicates that Dau produces a characteristic in-source fragmentation pattern. The main fragmentation pathway for daunomycin is the cleavage of the glycosidic bond, with the charge residing either on the aglycon part or on the sugar moiety, which co-occurs with secondary dissociation mechanisms for each fragment [16]. Furthermore, daunomycin conjugates also show extensive fragmentation under the standard ESI-MS workflow that uses slightly acidic solvents [14,17,18,19]. When the drug moiety is attached via oxime bond to the tumor targeting peptides, the cleavage of the sugar moiety from the daunomycin is the most prominent fragmentation pathway [14]. The same sugar loss fragmentation is pronounced during higher energy collision-induced dissociation (higher-energy C-trap dissociation, HCD) MS/MS analysis, thus suppressing the fragmentations of other types [15]. Consequently, MS and MS/MS analysis of daunomycin-bioconjugates results in a complex spectrum containing a mixture of daunomycin related fragments, generally hampering unambiguous assignment, accurate purity estimation and definitive structural identification. 

Different strategies, including the electron transfer dissociation (ETD) [20] and the electron transfer/higher energy collisional dissociation (EThcD) [21] are the most widely adopted fragmentation techniques often surpassing the sequence coverage of the HCD method. Thus, these methods hold great potential for enhanced characterization of peptide-based samples. Besides ESI, matrix-assisted laser desorption/ionization (MALDI) coupled to a tandem time-of-flight analyzer (TOF/TOF) can also provide highly sensitive peptide sequence information [22]. 

In the current study we focused on the structural identification of daunomycin-peptide conjugates using high resolution mass spectrometry and the above-mentioned MS/MS techniques aiming at high-coverage for the comprehensive characterization of PDCs. For this purpose, new daunomycin conjugates were synthesized containing α-MSH analogs as tumor targeting peptides. To investigate the influence of structural elements on the fragmentation, targeting peptides of different lengths were used and daunomycin was conjugated either to the *N*-terminus or to the lysine side chain of the peptides. We expected that the position of the daunomycin in structural isomers might lead to distinct MS/MS fragmentation patterns allowing confident assignment of the conjugation sites. 

## 2. Results and Discussion

### 2.1. Synthesis

Four α-MSH-daunomycin bioconjugates were chosen as model compounds to investigate the most optimal activation technique for the structural characterization of daunomycin-based bioconjugates and to illustrate possible differences in MS/MS fragmentation patterns. The possibility of differentiation between structural isomers was investigated, by incorporating Dau either to the *N*-terminus or to the side chain of Lys via oxime linkage by using aminooxyacetic acid (Aoa). The two underivatized peptides were used as references with known MS/MS fragmentation behaviors.

**Scheme 1:**
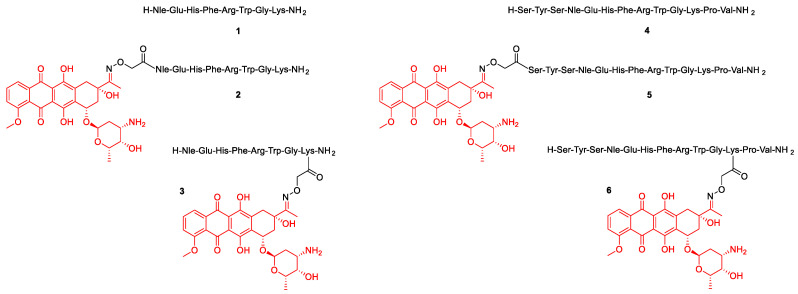
Molecular structure of the α-MSH peptides and bioconjugates.

The α-MSH peptide analogs **1** and **4** were synthetized by solid-phase peptide synthesis using the Fmoc/*t*Bu strategy. The Boc-Aoa-OH was coupled to the *N*-terminus (conjugates **2**, and **5**) or to the ε-amino group of ^8^Lys and ^11^Lys, respectively (conjugates **3**, and **6**). Dau was conjugated to the purified aminooxy moiety containing peptides by oxime bond formation in solution (0.2 M ammonium acetate buffer, pH 5.2) [23]. All bioconjugates and their reference peptides were purified by RP-HPLC. The structures of the peptides and bioconjugates and their analytical properties are summarized in Scheme 1 and Table 1. A representative scheme for the synthesis of peptide **1** and conjugates **2** and **3** is shown in the Supporting Information (Scheme S1). Compounds **4**–**6** have been prepared in the same way.

### 2.2. HCD Experiments

**Figure 1:**
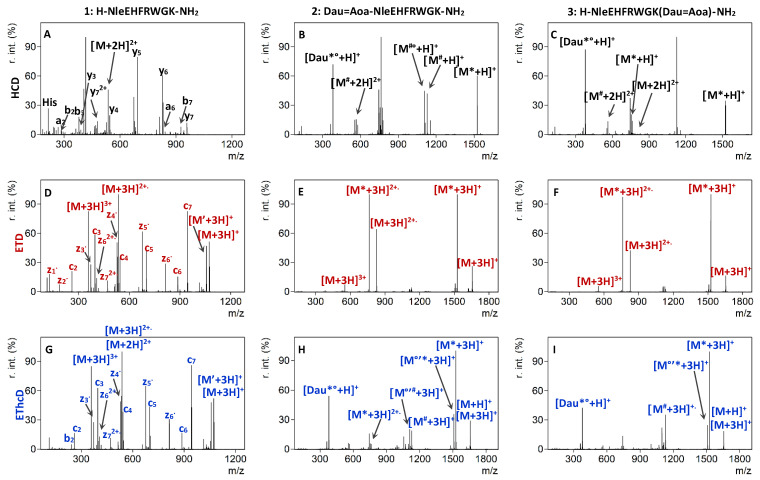
HCD MS/MS spectra of the doubly protonated peptide 1 (**A**) at 30% NCE and conjugates 2 (**B**), and 3 (**C**) at 20% NCE. ETD MS/MS spectra of the triply protonated 1 (**D**), 2 (**E**), and 3 (**F**). EThcD MS/MS spectra of the triply protonated 1 (**G**) at 10% NCE, and 2 (**H**) and 3 (**I**) at 40% NCE. Neutral losses are depicted as follows: * = sugar, ’ = NH_3_, ° = H_2_O, # = Dau + sugar.

The HCD MS/MS spectra of the unmodified doubly charged peptides **1** and **4** exhibited significant peptide backbone fragmentation producing singly and doubly protonated *b* and *y* type fragments accompanied with neutral losses of water and ammonia. As expected, complete *b* and *y* ion series can be detected in the HCD spectrum of the 8-mer peptide **1** when the normalized collision energy was set between 20% and 40% (Figure 1A). 

**Scheme 2:**
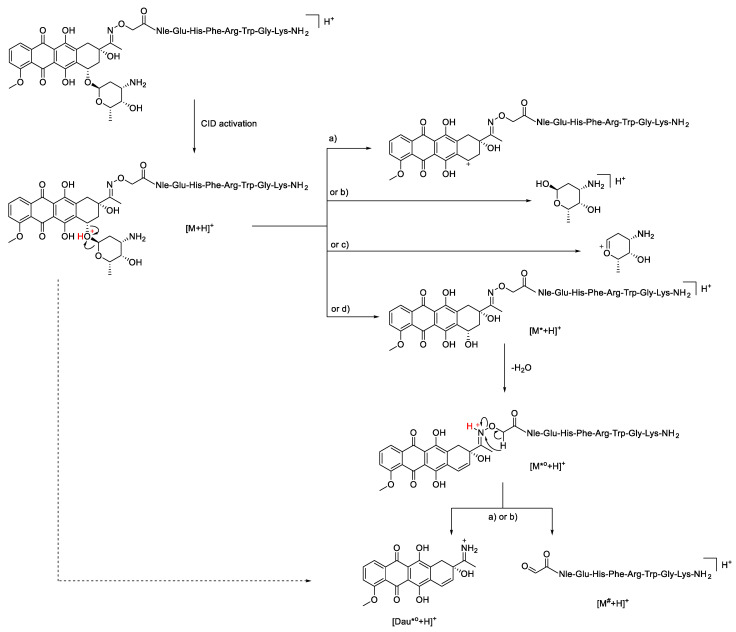
Cleavage mechanism of the glycosidic bond according to the literature [25], and the fragmentation scheme proposed herein for the loss of Dau moiety by the breakage of the oxime bond. Corresponding neutral fragments formed upon the cleavages of the glycosidic or oxime bonds are present but are not depicted for the ease of readability. Neutral losses are marked as follows: * = sugar, ° = H_2_O, # = Dau + sugar.

The same behavior is observed in the case of the 13-mer peptide **4** between 30% and 40% (Figure S1A). In contrast, in the HCD MS/MS spectrum of the doubly charged daunomycin containing conjugates **2** and **3**, the most intense ions correspond to the fragmentation of the anthracycline drug [16], while purely peptide-related fragments could not be detected at low NCE values, up to 20% (Figure 1B,C). The predominant fragmentation pathway for all daunomycin-peptide conjugates was the loss of the amino sugar of the daunomycin. After cleavage of the *O*-glycosidic bond between the aglycone part and the daunosamine sugar, initiated by the protonation of the oxygen, the charge can remain either on the sugar portion or on the aglycone moiety, which can subsequently lose a water molecule (Scheme 2). Protonated sugar-lost conjugates are marked with [M*+H]^+^, whereas the subsequent water loss is depicted as [M*°+H]^+^. These results correlate with our previously published data, where only the protonated sugar and the protonated, but charge-reduced sugar-lost conjugate could be detected in the HCD MS/MS spectrum of a doubly protonated Dau = Aoa-SKAAKN-OH conjugate at 10 eV collision energy using a Thermo Scientific Q Exactive Focus mass spectrometer [15]. 

The N-O bond of the oxime moiety is also prone to fragmentation leading to the loss of daunomycin drug from the conjugate. Our proposed mechanism for the cleavage of the N-O bond is the acyclic β-hydrogen rearrangement [24]. Scheme 2 shows the possible route leading to the formation of the imine group containing protonated unsaturated aglycone ([Dau*°+H]), which can be detected with *m*/*z* 380.1129, together with protonated oxoacetylated peptide ions marked as [M^#^+H]^+^ (see also Figure 1B,C). The exact same fragmentation pattern characterizes the low collision energy HCD MS/MS spectra of conjugates **5** and **6** (Figure S1B,C).

It was observed, that besides the cleavage of the sugar of the daunomycin moiety, the fragmentation of the peptide backbone also occurred when a higher HCD energy was used. Increasing the collision energy to 25–35% NCE led to the appearance of a *y*_4_, *y*_5_, *y*_6_ and *y*_7_ ions in the tandem mass spectrum of the doubly charged conjugate **2**, while *b*_3_, *b*_5_, *b*_6_ and *b*_7_ ions could be detected in the case of conjugate **3** (Figure S2). Likewise, uncomplete *y* and *b* ion series could be detected in conjugates **5** and **6**, containing the longer homing peptide. Thus, no direct correlation could be established between the length of the peptide chain and its effect on the fragmentation. Remarkably, the intensity and abundance of peptide sequence related fragments remained inferior to the cleavage of the daunosamine moiety. 

**Figure 2:**
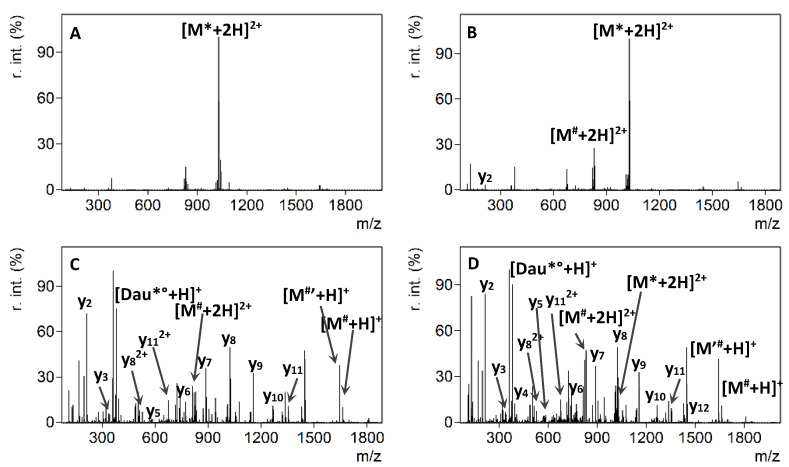
HCD MS/MS spectra of the doubly and triply protonated conjugate 5 at 20% NCE (**A**,**B**, respectively) and at 30% NCE (**C**,**D**, respectively). Neutral losses are depicted as follows: * = sugar, ’ = NH_3_, ° = H_2_O, # = Dau + sugar.

The observation that higher charge states allow for easy fragmentation has initiated the investigation of the fragmentation behaviors of triply protonated conjugates. Although doubly protonated unmodified peptides are mainly in the focus of HCD fragmentation techniques, conjugation of the peptides with daunomycin increased both the molecular weight and the basicity of the conjugates. Therefore, we hypothesized that selection of higher charge states may be required for the effective fragmentation of conjugates containing daunomycin modification. Hence, we used higher collision energies for the MS/MS fragmentation of triply protonated conjugates **2**, **3**, **5** and **6**. A comparative example can be seen in Figure 2. The HCD MS/MS spectrum of the doubly protonated conjugate **5** at 20% contained only the doubly protonated sugar lost molecule (Figure 2A), while, on the other hand, the tandem mass spectrum of the triply protonated conjugate **5** at 30% NCE showed a complete *y* series, while *b* and *a* ions were not observed (Figure 2D). These results demonstrated that an appropriate selection of the precursor charge state and HCD energy can ultimately result in efficient peptide backbone fragmentation of the conjugates. Apparently, higher energies and higher charge states were required to “mobilize” the protons to facilitate charge-directed backbone cleavages [26]. These protons were sequestered not only at the basic side chain containing amino acids, such as Arg or Lys, but most probably also at daunosamine sugar part.

Based on the results of the above experiments we concluded that efficient HCD fragmentation depends on the selected charge state and HCD energy. Doubly protonated control peptides fragment more easily at a wide NCE% range. However, higher energy onset and the selection of higher charge state precursors are required for the fragmentation of the daunomycin conjugates and for the enhanced sequence coverage (Table 2).

### 2.3. ETD and EThcD Experiments

Electron-transfer dissociation is the method of choice for the identification of long and highly charged peptides. ETD outperforms CID or HCD for charge states higher than 2 and induces more backbone cleavages, thus increasing the peptide sequence coverage [27]. ETD is better than other similar methods, because it preserves post-translational modifications (PTMs), which are often labile after CID, thereby making available all relevant sequence information [28]. Therefore, triply charged ions were selected for ETD fragmentation in our experiments. In the case of peptides **1** and **4**, ETD MS/MS resulted in a virtually complete or almost complete sequence coverage. For peptide **1**, all seven N-C_α_ bonds were cleaved generating a complete series of *c* and *z*^•^-type fragment ions (Figure 1D). Surprisingly, conjugation of daunomycin to the α-MSH tumor homing peptides drastically inhibited the N-C_α_ backbone cleavage following electron transfer by triply charged conjugate ions. The ETD MS/MS spectra of conjugates **2** and **3** were poor, containing only four intense fragment ions, namely the [M+3H]^3+^ precursor and the [M+3H]^2+•^ charge-reduced species, together with sugar-lost conjugates [M*+3H]^+^ and [M*+3H]^2+•^ (Figure 1E,F). The backbone fragmentation was incomplete regardless of the length of the targeting peptide or the position of the drug in the bioconjugates (Figure S1E,F).

The presence of specific functional moieties within a peptide sequence can limit the fragmentation of the peptide backbone in ETD or electron-capture dissociation (ECD). This phenomenon can be explained by the “electron predator” effect. This model, first described by Sohn et al. [29], proposes the presence of a functional group (i.e., 3-nitrobenzyl [29] or 3-nitrotyrozyl [30,31] groups) with high electron affinity (EA), that captures electrons and forms a stable radical intermediate inhibiting peptide backbone fragmentation in ETD or ECD. These disadvantageous features limit the application of these two techniques for peptide identification containing such modifications. In general, substituents with EA > 1.00 eV are mentioned as “electron predator” that completely prevent N-C_α_ backbone cleavage [29]. Anthracyclines contain the 9,10-anthraquinone nucleus which has high EA (1.59 eV) [32], as well as undergo gas-phase reactions indicative of their high EAs [33]. Furthermore, anthracycline antibiotics are involved in single electron transport mechanisms in microsomes [34]. Considering that anthracycline drugs, such as daunomycin, fulfill the beforementioned high EA criteria and produce an incomplete backbone fragmentation, as was observed herein in the case of all four conjugates, this class of drugs can be considered as new members of the “electron predator” family.

Compared with HCD and ETD, the electron transfer and higher energy collisional dissociation (EThcD) technique provides a considerable increase in peptide backbone fragmentation of doubly charged peptides and enables a confident structure identification [21], including the localization of different PTMs [35,36,37]. Therefore, we anticipated that this method can be successfully applied for the analysis of daunomycin conjugates. Although the EThcD MS/MS spectrum of the triply charged peptide ions at 40% NCE produced numerous *b*, *c*, and *z*^•^ fragments, as well as charge-reduced precursor ions and several neutral losses (Figure 1G), the spectra of the triply charged conjugates **2** and **3**, measured with 40% NCE, were dominated by charge-reduced precursors, and sugar- and Dau-lost ions (Figure 1H,I). Similar to the results of ETD measurement, no peptide backbone fragmentation could be observed in the case of the daunomycin-peptide conjugates. Likewise, the transfer of electrons can be captured by the daunomycin conjugated either to the *N*-terminus or the Lys side chain of the peptides and the subsequent collisional activation halts the cleavage of the peptide chain. The MS/MS spectrum of peptide **4** also shows abundant fragmentation, whereas the spectra of conjugates **5** and **6** mainly contains losses of sugar and the Dau moiety, which was accompanied by ammonia loss of charge-reduced precursors (Figure S1H,I).

Based on the results of these experiments we concluded that none of the electron transfer-based techniques gives a reliable analytical characterization of the daunomycin conjugates. The ETD and EThcD MS/MS spectra of the selected conjugates did not contain beneficial information for interpreting the peptide sequence nor the position of the daunomycin. The fragmentation efficiency of α-MSH analogs and Dau-conjugates by HCD, ETD and EThcD activation methods is summarized in Table 2.

### 2.4. MALDI-TOF/TOF Experiments

Matrix-assisted laser desorption/ionization (MALDI) based tandem mass spectrometry has also been proved as a powerful tool in structural characterization of peptide and protein samples [22,38]. In MALDI-TOF/TOF, peptide fragmentation can be obtained by laser-induced dissociation (LID) or high-energy collision-induced dissociation (CID), known as real MS/MS techniques and in-source decay (ISD) as a pseudo-MS/MS technique [39]. Typically, in MS/MS mode singly charged precursor ions are selected and fragmented. Moreover, singly charged fragment ions are analyzed in this mode. These result in a simplified product ion spectrum and enable rapid interpretation as well as accurate sequencing. By comparison, MALDI-ISD involves a prompt fragmentation occurring in the MALDI ion source, and generates the cleavage of the peptide backbone bonds, thus, leading to sequence specific information [40].

**Figure 3:**
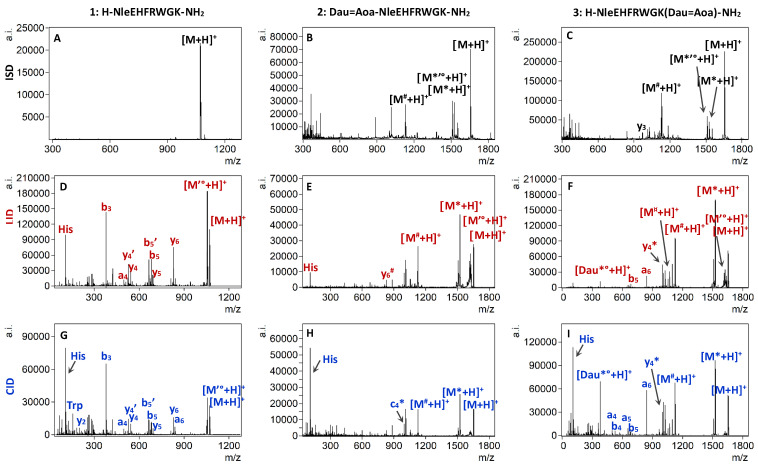
MALDI MS/MS spectra of the singly protonated compounds **1**, **2**, and **3**. (**A**–**C**) MALDI-ISD spectra, (**D**–**F**) MALDI-TOF/TOF LID spectra, (**G**–**I**) MALDI-TOF/TOF CID spectra. Neutral losses are depicted as follows: * = sugar, ’ = NH_3_, ° = H_2_O, # = Dau + sugar.

First, peptides and bioconjugates were fragmented by ISD (Figure 3A–C, Figure S3A–C respectively). All spectra contained abundant [M+H]^+^ ions and a few fragment ion peaks, because the efficiency of producing fragment ions via the MALDI-ISD mode is relatively low for short peptides [41]. The MALDI-ISD spectra of peptides **1** and **4** contains a very small number of fragments with low abundance. Similarly to HCD, in the case of all conjugates the favored fragmentation pathways include the neutral loss of the sugar moiety from the daunomycin, as well as the loss of the daunomycin molecule (Scheme 2), accompanied by additional losses of ammonia and water from these fragments (Figure 3B,C, and Figure S3B,C). The ISD dissociation of daunomycin conjugates also yielded very few peptide-related fragments and a large portion of unassigned peaks in the lower mass region (300–500 *m*/*z*). In our experiments, the DHB matrix was used, which favors the production of *a*-, *b*-, and *y*-ions via the thermal fragmentation pathway due to its low proton affinity [40].

Besides different neutral losses, the LID fragmentation of peptides **1** and **4** yielded a mixture of incomplete *a*, *b* and *y* series, and the sequence coverage for the 13-mer peptide **4** being greater than for the 8-mer analog **1** (Figure 3D, and Figure S3D). The MALDI-LID-MS/MS spectra which were acquired from the singly protonated precursors of conjugates **2** and **3** are shown in Figure 3E,F, respectively, whereas those of conjugates **5** and **6** are shown in Figure S3E,F, respectively. Although the sugar-lost conjugate was the most dominant fragment ion in the MS/MS spectra of all daunomycin conjugates, fragmentation of the peptide backbone amide bonds was also detectable with various intensities. The His immonium ion and the *y*_6_^#^ fragment were recorded for conjugate **2**, where Dau is conjugated to the *N*-terminus, while *b*_3_, *b*_5_, *a*_6_, and *y*_4_* fragment ions were observed for conjugate **3**, in which Dau is attached to the Lys side chain (neutral losses: # = Dau + sugar, * = sugar). Higher mass *a*-type fragment ions (*a*_8_, *a*_12_ and *a*_9_, *a*_12_, respectively) gained intensity when the conjugates contained the longer 13-mer targeting peptide (i.e., conjugates **5**, and **6**). 

These results revealed a clear dependency of the peptide backbone fragmentation on the position of the daunomycin in the conjugates. Fragment ions, which were present when Dau was conjugated on the *N*-terminus were, however, not detectable when the drug was attached to the Lys, and vice versa. In addition, the length of the peptide chain also affected the MALDI-LID fragmentation behavior of daunomycin conjugates. However, the abundance and intensity of the targeting peptide-related fragments was still low compared to the intensity of fragment ions corresponding to the decomposition of the anthracycline drug. Therefore, the present data were insufficient to determine the conjugations sites or identify the sequence. 

The CID spectrum of peptide **1** using N_2_ as collision gas contained more fragment ions compared to the LID spectrum because of their different fragmentation pattern (Figure 3G). We also observed that the fragment ions’ signal intensity and abundance was clearly shifted to the lower third of the mass region according to the result of CID activation for peptide **4** (Figure S3G). This effect is presumably due to multiple collision and fragmentation events resulting in fewer mass fragments. The same behavior was reported by Macht et al. in a comparative study of the LID and CID fragmentation processes for ACTH (1–17), a structural analog of peptide **4**, and other peptides [42]. 

Remarkably, the MALDI-CID activation of the daunomycin-conjugate precursor ions produced a more informative product ion spectrum, some characteristic peptide-sequence-related fragments had comparable intensity to those of sugar and Dau loss fragments (Figure 3H,I, Figure S3H,I. Most notably, the relative intensity of specific *a*-type ions showed a significant increase compared to the respective LID spectra. By comparing the MALDI-CID spectra of conjugates **2** with **3** and **5** with **6**, it can be observed that the fragmentation pattern was influenced by the position of the daunomycin. In fact, if Dau was conjugated to the *N*-terminus (conjugates **2**, and **5**), several unassigned signals as well as middle-sized *y* and *b*-type fragment ions are observed in the spectrum. These low intensity fragments are most probably formed due to the MALDI laser-induced excitation of the Dau at the *N*-terminus, which directed the fragmentation to the middle of the peptide chain. A characteristic *a*-fragment ion (*a*_8_) was only present when the longer homing peptide was used (Figure S3H), indicating that the effect of this phenomenon may be dependent on the length of the peptide chain. In contrast, besides small fragments, a very intense *a*-type ion was detectable in the MALDI-CID spectrum of conjugates **3** and **6**, in which Dau was conjugated to the ε-amino group of the Lys. The *a*_6_ and *a*_9_ ions (for conjugates **3** and **6**, respectively) were both produced by the breakage of the α-carbon to carbonyl-carbon bond (CH-CO) between the Trp and Gly. Preferential cleavage was also found at *N*-terminal to Gly by Khatun et al. resulting in *y* fragments [43]. The fragmentation efficiency of α-MSH analogs and bioconjugates by MALDI MS/MS is summarized in Table 3.

**Scheme 3:**
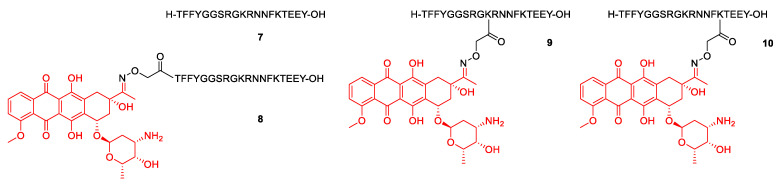
Molecular structure of the Angiopep-2 (**7**) and Angiopep-2-daunomycin conjugates **8**, **9** and **10**.

To verify whether the characteristic fragment ions were indeed generated through the high-energy CID process, we selected structurally more complex peptide conjugates and subjected them to MALDI MS/MS analysis. The 19-mer Angiopep-2 peptide (**7**, *H*-TFFYGGSRGKRNNFKTEEY-*OH*, Scheme 3), which is a specific ligand of the low density lipoprotein 1 (LRP-1) [44], contains three potential sites for conjugation. Daunomycin can be conjugated either to the *N*-terminus of the peptide or to one of the lysine side chains in the sequence (^10^Lys or ^15^Lys) resulting in three daunomycin conjugate isomers (**8**–**10**, Scheme 3). 

**Figure 4:**
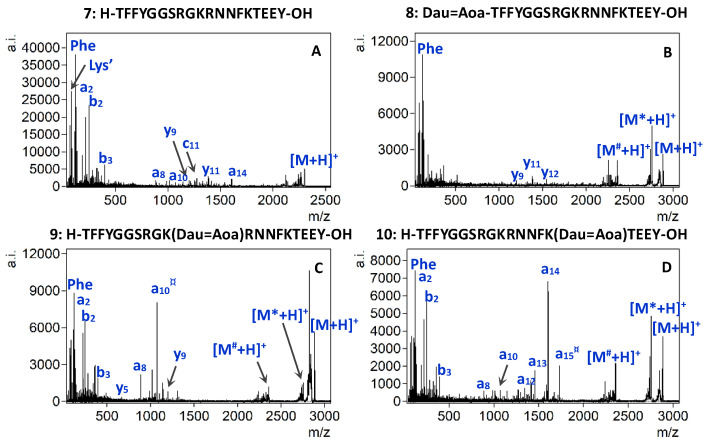
MALDI-CID-MS/MS spectra of the compounds **7** (**A**), **8** (**B**), **9** (**C**), and **10** (**D**). Neutral losses are depicted as follows: * = sugar, ’ = NH_3_, ° = H_2_O, # = Dau + sugar, ¤ = Dau + sugar + Aoa.

Figure 4 shows the MALDI-CID spectra of Angiopep-2 (**7**) and conjugates **8**, **9** and **10** acquired using identical conditions as before. In general, neutral loss of the sugar and Dau moiety was detectable for all three conjugates. Furthermore, the fragmentation pathways of the three conjugates significantly differed from each other. The comparison of the MALDI-CID spectra confirmed a different fragmentation behavior for conjugates containing *N*-terminal and Lys modification. In the case of conjugate **8**, which contained Dau at its *N*-terminus, the peptide backbone fragmentation was limited. Moreover, the *y*-type ions were present with low abundance and intensity. In addition, immonium ions were obtained with slightly higher intensities (Figure 4B). While the iminium ion region of the tandem mass spectrum of conjugates **9** and **10** was similar to that of conjugate **8**, lower mass ions (i.e., *a*_2_, *b*_2_ and *b*_3_) showed increased intensity and specific fragment ions gained significant abundance in the middle mass region when Dau was conjugated to one of the Lys side chains (Figure 4C and 4D, respectively). Furthermore, a comparison of the tandem mass spectrum of **9** with **10**, in which Dau was conjugated to the ε-amino group of the ^10^Lys or ^15^Lys, revealed a considerable difference in terms of the dominant fragment ions present in each spectrum. The *a*-type fragment ions, containing or in the vicinity of the modified Lys, were the most intense fragments. While the *a*_10_^¤^ was the dominant fragment ion in the tandem mass spectrum of conjugate **9**, *a*_14_ ion became the most abundant fragment in the MALDI-CID spectrum of conjugate **10**.

The presence of the highly intense *a*_10_^¤^ or *a*_14_ fragments, together with the absence of these in the MS/MS spectrum of the respective conjugate isomer pair, ensured unambiguous localization of the conjugation site. This information allows a reliable analytical characterization of large peptide-drug conjugates that contain multiple conjugation sites. 

## 3. Materials and Methods

### 3.1. Synthesis of the Daunomycin-Peptide Conjugates

All amino acid derivatives, the Rink-amide MBHA resin, Wang resin, *N*,*N*′-diisopropylcarbodiimide (DIC), and trifluoroacetic acid (TFA) were purchased from Iris Biotech GmbH (Marktredwitz, Germany). Boc-aminooxyacetic acid (Boc-Aoa-OH), 1-hydroxybenzotriazole hydrate (HOBt), triisopropylsilane (TIS), and hydrazine hydrate were obtained from Sigma Aldrich Kft. (Budapest, Hungary), 1,8-diazabicyclo[5.4.0]undec-7-ene (DBU) was acquired from TCI Europe N.V. (Zwijndrecht, Belgium), and piperidine was purchased from Molar Chemicals Kft (Budapest, Hungary).

The peptides **1** and **4** were manually synthetized using the standard Fmoc/*t*Bu strategy on Rink-Amide MBHA resin (0.69 mmol g^−1^). For the synthesis of Dau containing conjugates **2** and **5**, the *N*-terminal Fmoc group was cleaved with 2% piperidine + 2% DBU in DMF (2 + 2 + 5 + 10 min), while for the synthesis of conjugates **3** and **6**, the Lys side chain protecting group (Dde) was selectively cleaved with 2% hydrazine hydrate (6 × 2 min). Boc-Aoa-OH was coupled either to the *N*-terminus or to the ε-amino group of the Lys using the standard Fmoc/*t*Bu protocol. Peptides were cleaved from the resin with 95% TFA, 2.5% water, 2.5% TIS (*v*/*v*/*v*%) for 2 h at room temperature. The crude peptides were lyophilized, purified by RP-HPLC, and the pure Aoa-derivatized fractions were immediately used for the next step. Daunomycin was conjugated to the peptides in solution (0.2 M ammonium acetate buffer, pH 5.2) [23]. The mixture was stirred for 2 days and the conjugates **2**, **3**, **5**, and **6** were obtained after RP-HPLC purification.

Peptide **7** was synthetized using the standard Fmoc/*t*Bu strategy on Wang resin (0.45 mmol g^−1^). For the synthesis of **9** and **10**, Fmoc-Lys (Dde)-OH was incorporated in positions **10** and **15**, respectively. The following steps were performed as described above.

### 3.2. Instruments

Prior to HCD, ETD and EThcD measurements, freeze-dried samples were diluted to 10 µM final concentration with acetonitrile-water (1:1, *v*/*v*) containing 0.1 *v*/*v*% formic acid. Solutions were directly injected into the electrospray source of an Orbitrap Fusion Tribrid instrument (Thermo Scientific, Waltham, MA, USA) at a flow rate of 5 μL min^−1^. Samples were ionized with 3500 V constant current in positive mode. The flow rate of the sheath gas was approximately 0.6 L min^−1^ and the flow rate of the aux gas was approximately 2.5 L min^−1^. The vaporizer temperature was set to 30 °C and the temperature of the ion transfer tube was 300 °C. Resolution was set to 120,000. An isolation width of 2 *m*/*z* was applied for MS/MS. For ETD and EThcD experiments, the ion activation time was set to 50 ms. Normalized collision energies (NCE) were set between 5 and 45% for HCD and EThcD.

Prior to MALDI-TOF/TOF measurements, samples were dissolved in acetonitrile-water (1:1, *v*/*v*) containing 0.1% TFA. 2,5-dihydroxybenzoic acid (DHB, 100 mg/mL, acetonitrile-water (1:1, *v*/*v*%) with 0.1% TFA) was used as matrix. The matrix and the sample were mixed in the ratio of 1:1 and 0.25 μL was deposited onto the stainless steel 384-well sample plate and allowed to air-dry. MALDI-TOF/TOF measurements were carried out with a Bruker Autoflex Speed mass spectrometer (Bruker Daltonics, Bremen, Germany) operating in the reflectron positive ion mode. For ISD measurements 19 kV (ion source voltage 1), 16.7 kV (ion source voltage 2), 21 kV (reflector voltage 1) and 9.65 kV (reflector voltage 2) voltages were used. For LIFT measurements 6 kV (ion source voltage 1), 5.25 kV (ion source voltage 2), 27 kV (reflector voltage 1) and 11.65 kV (reflector voltage 2) voltages were applied. The solid phase laser (355 nm) was operated at 500 Hz and 2000 shots were summed. The spectra were externally calibrated using adequate standard peptides (Bruker Daltonics, Bremen, Germany). The CID experiments were performed applying nitrogen as collision gas at a pressure level of 6 × 10^−6^ mbar in the source region (MALDI-CID). The mass resolution of the MALDI-CID measurements was 1400 at *m*/*z* 400 and 3300 at *m*/*z* 2100, determined by the spectra of sample **7**.

All spectra were analyzed with Xcalibur (version 3.1.66.10, Thermo Scientific, Waltham, MA, USA) and/or Mmass [45].

## 4. Conclusions

Sequencing and identifying the structures of daunomycin-peptide conjugates are considerably challenging owing to their complex structures and the prominent fragmentation of this anthracycline drug. In a previous study, we demonstrated that in-source and HCD fragmentation of daunomycin conjugates leads to the loss of the sugar moiety from the daunomycin, resulting in complex spectra, whereas the peaks of intact protonated conjugates are of low intensity.

In the present work, we aimed to identify an adequate MS/MS method that provided high sequence coverage for a reliable structural identification of daunomycin conjugates. We found that daunomycin-related fragmentation is still the major fragmentation pathway using the most common fragmentation techniques, namely, HCD, ETD and EThcD. Conjugation of the peptides with daunomycin increased both the molecular weight and the gas-phase basicity of the conjugates. Such chemical modification greatly influenced the fragmentation efficiency of the bioconjugates, higher energies and higher charge states were required to “mobilize” the protons and facilitate charge-directed backbone cleavages than for the unmodified peptides. Based on these results, an appropriate selection of the precursor charge state and HCD energy can provide a complete sequence coverage and facilitate the conjugation site assignment.

We have also shown that peptide backbone fragmentation was inhibited in these conjugates using ETD or EThcD methods, most possibly due to the “electron predator” effect of the daunomycin.

MALDI-TOF/TOF analysis of daunomycin bioconjugates yielded structural information about the targeting peptide and allowed discrimination between different drug conjugation sites even in the case of large and complex molecules. When used along with collisional activation, the MALDI-MS/MS spectra of the conjugates were dominated by fragments related to characteristic peptide sequences aside from the facile sugar and daunomycin lost fragments. The presence of intense a-ions, which corresponded to the peptide backbone cleavage at or within the vicinity of Lys residues that contained the modifications, improved the localization of side chain conjugations. However, MALDI-TOF/TOF provided an incomplete sequence coverage.

We envision that MALDI-CID-MS/MS will be the method of choice for structure identification and comprehensive characterization of peptide-drug conjugates containing anthraquinone, quinoline or other polyheterocyclic analogs as payloads.

## Figures and Tables

**Table 1:** Structural and analytical properties of the α-MSH analogs and bioconjugates.

	Compound	*m*/*z* Calc. for [M+2H]^2+^	*m*/*z* Meas. ^1^ for [M+2H]^2+^	Deviation (ppm)	R_t_ (min) ^2^
**1**	H-NleEHFRWGK-NH_2_	536.2960	536.2954	1.1	6.0
**2**	Dau = Aoa-NleEHFRWGK-NH_2_	827.3884	827.3876	1.0	8.4
**3**	H-NleEHFRWGK(Dau = Aoa)-NH_2_	827.3884	827.3875	1.1	8.1
**4**	H-SYSNleEHFRWGKPV-NH_2_	802.9202	802.9192	1.2	7.2
**5**	Dau = Aoa-SYSNleEHFRWGKPV-NH_2_	1094.0127	1094.0114	1.2	8.6
**6**	H-SYSNleEHFRWGK(Dau = Aoa)PV-NH_2_	1094.0127	1094.0115	1.1	8.6

^1^ ESI-MS data measured on a Thermo Scientific Q Exactive Focus mass spectrometer. ^2^ Analytical UHPLC was performed using a Waters Acquity UPLC BEH C18 1.7 μm column (2.1 × 50 mm), at 40 °C; eluent A: water + 0.1% formic acid, eluent B: 80% acetonitrile + 0.1% formic acid. The following gradient was used: 0 min 2% B, 1 min 2% B, 17 min 100% B, 17.5 min 100% B, 18 min 2% B, 21 min 2% B. The flow rate was 300 μL min^−1^. Dau stands for daunomycin, Aoa stands for aminooxyacetic acid, Nle stands for norleucine.

**Table 2:** Fragmentation efficiency of the α-MSH analogs and bioconjugates by HCD, ETD and EThcD fragmentation techniques.

Compound	*z*	Low E HCD (NCE ≤ 20%)	High E HCD (NCE ≥ 25%)	ETD	EThcD
**1**; (**4**)	2	+; (−)	+ +; (+ + +)	+ +	+ + ^a^
	3	+ + +; (+ +)	+ + +; (+ +)	+ + +; (+ +)	+ + + (+ +) ^a^
**2**; (**5**) Dau at *N*-terminus	2	−	+ +	−	+; (+ +) ^b^
	3	+; (−)	+ +	−	−; (+) ^b^
**3**; (**6**) Dau at Lys	2	−	+; (+ +)	−	− ^c^
	3	−	+; (+ +)	−	− ^d^

Symbols: −: no peptide sequence related fragments; +: the sequence coverage is 10–30%, where the relative intensity of the fragments is ≥5%; + +: the sequence coverage is 31–60%, where the relative intensity of the fragments is ≥5%; + + +: the sequence coverage is > 61%, where the relative intensity of the fragments is ≥5%; ^a^ NCE = 10%, ^b^ NCE = 40%, ^c^ NCE = 30%, ^d^ NCE = 35%. Values for the compounds **4**, **5** and **6** are in brackets.

**Table 3:** Fragmentation efficiency of singly protonated α-MSH analogs and bioconjugates by MALDI MS/MS.

Compound	ISD	LID	CID
**1**; (**4**)	−	+ +; (+ + +)	+ + +; (+ +)
**2**; (**5**) Dau at *N*-terminus	−	+	+
**3**; (**6**) Dau at Lys	−	+ +; (+)	+ +; (+)

Symbols: −: no peptide sequence related fragments; +: the sequence coverage is 10–30%, where the relative intensity of the fragments is ≥5%; + +: the sequence coverage is 31–60%, where the relative intensity of the fragments is ≥5%; + + +: the sequence coverage is ≥61%, where the relative intensity of the fragments is ≥5%. Values for the compounds 4, and 6 are in brackets.

## Data Availability

Data is contained within the article or Supplementary Material.

## Supplementary Materials

The following are available online at https://www.mdpi.com/1422-0067/22/4/1648/s1, Scheme S1: Synthesis of the peptide **1** and Daunomycin-α-MSH conjugates **2** and **3**. Figure S1: HCD MS/MS spectra of doubly protonated **4** (A) at 30% NCE, **5** (B) and **6** (C) at 20% NCE. ETD MS/MS spectra of triply protonated **4** (D), **5** (E) and **6** (F). EThcD MS/MS spectra of triply protonated **4** (G) at 10% NCE, **5** (H) at 40% NCE and **6** (I) at 30% NCE, Figure S2: HCD MS/MS spectra of triply protonated **5** (A) and **6** (B) at 20% NCE, Figure S3: MALDI MS/MS spectra of the singly protonated compounds **4**, **5**, and **6**. A, B, C: MALDI-ISD spectra, D, E, F: MALDI-TOF/TOF LID spectra, G, H, I: MALDI-TOF/TOF CID spectra. Table S1: Structural and analytical properties of the Angiopep-2 and Angiopep-2-bioconjugates. MS spectra of the compounds **1**–**10**.
