# Supplementary material for: Structural Characterization of Daunomycin-Peptide Conjugates by Various Tandem Mass Spectrometric Techniques
Source: Int J Mol Sci. 2021 Feb 6;22(4):1648. doi: 10.3390/ijms22041648 (PMC7914584; doi:10.3390/ijms22041648)
Supplement: Supplementary file 1 [file ijms-22-01648-s001.pdf]

Structural Characterization of Daunomycin-peptide Conjugates by Various Tandem Mass Spectrometric Techniques

Adina Borbély [1], Lilla Pethő [2], Ildikó Szabó [2], Mohammed Al-Majidi [1,3], Arnold Steckel [1,3], Tibor Nagy [4], Sándor Kéki [4], Gergő Kalló [5], Éva Csősz [5], Gábor Mező [2,6] and Gitta Schlosser [1,*]

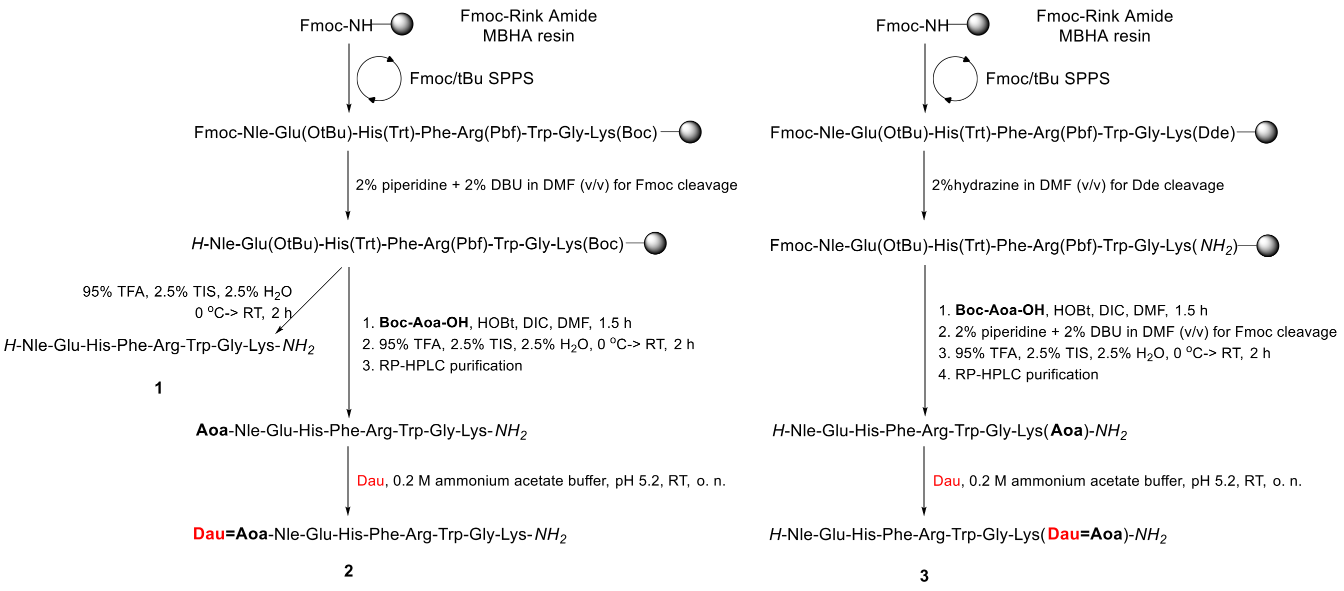

Scheme S1: Synthesis of the peptide **1** and Daunomycin-α-MSH conjugates **2** and **3**.

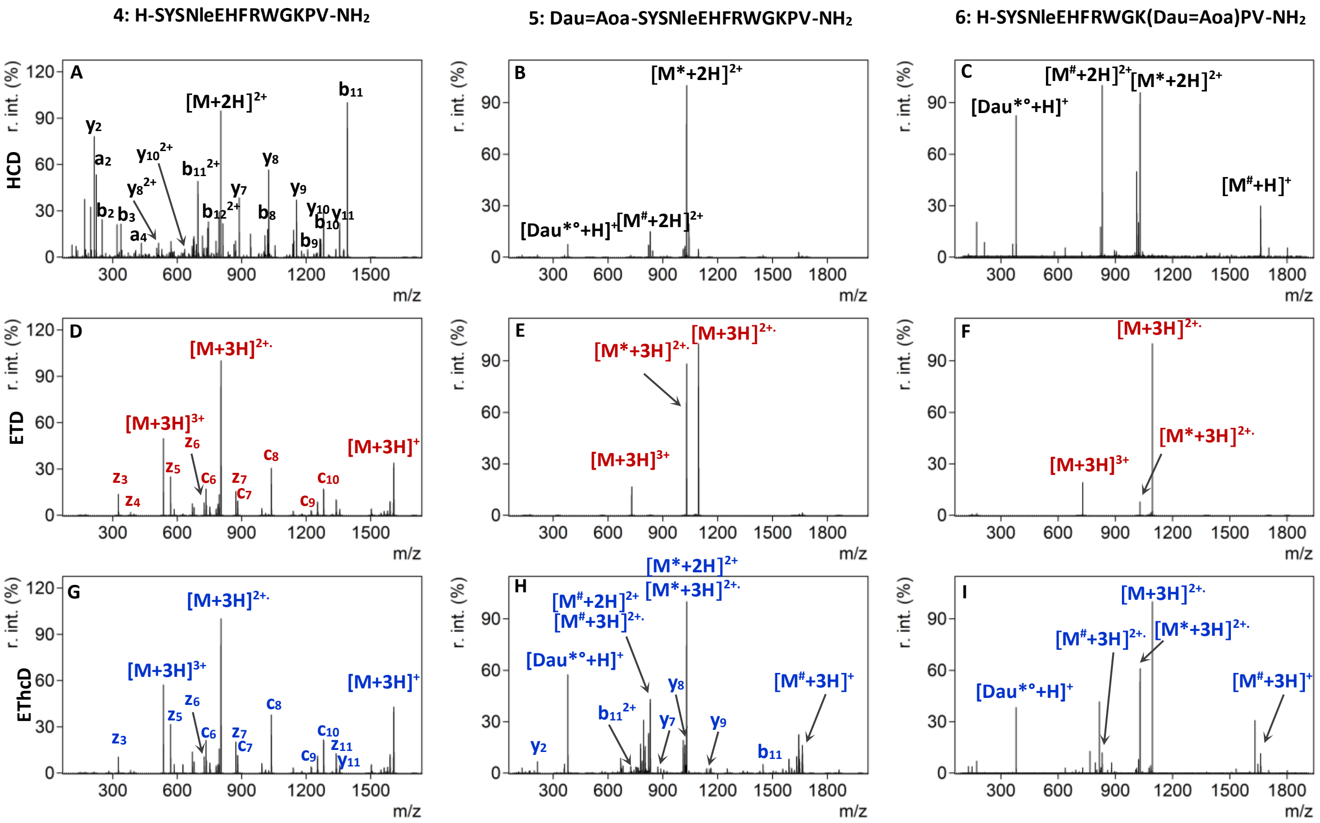

Figure S1: HCD MS/MS spectra of doubly protonated **4** (A) at 30% NCE, **5** (B) and **6** (C) at 20% NCE. ETD MS/MS spectra of triply protonated **4** (D), **5** (E) and **6** (F). EThcD MS/MS spectra of triply protonated **4** (G) at 10% NCE, **5** (H) at 40% NCE and **6** (I) at 30% NCE. Neutral losses are depicted as follows: °=H_2O, *=sugar, #=Dau+sugar. For the ease of labeling, neutral losses of water or ammonia from precursors are not depicted.

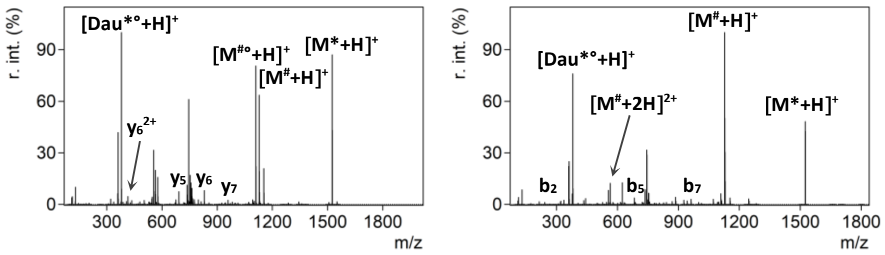

Figure S2: HCD MS/MS spectra of triply protonated **2** (A) and **3** (B) at 25% NCE.

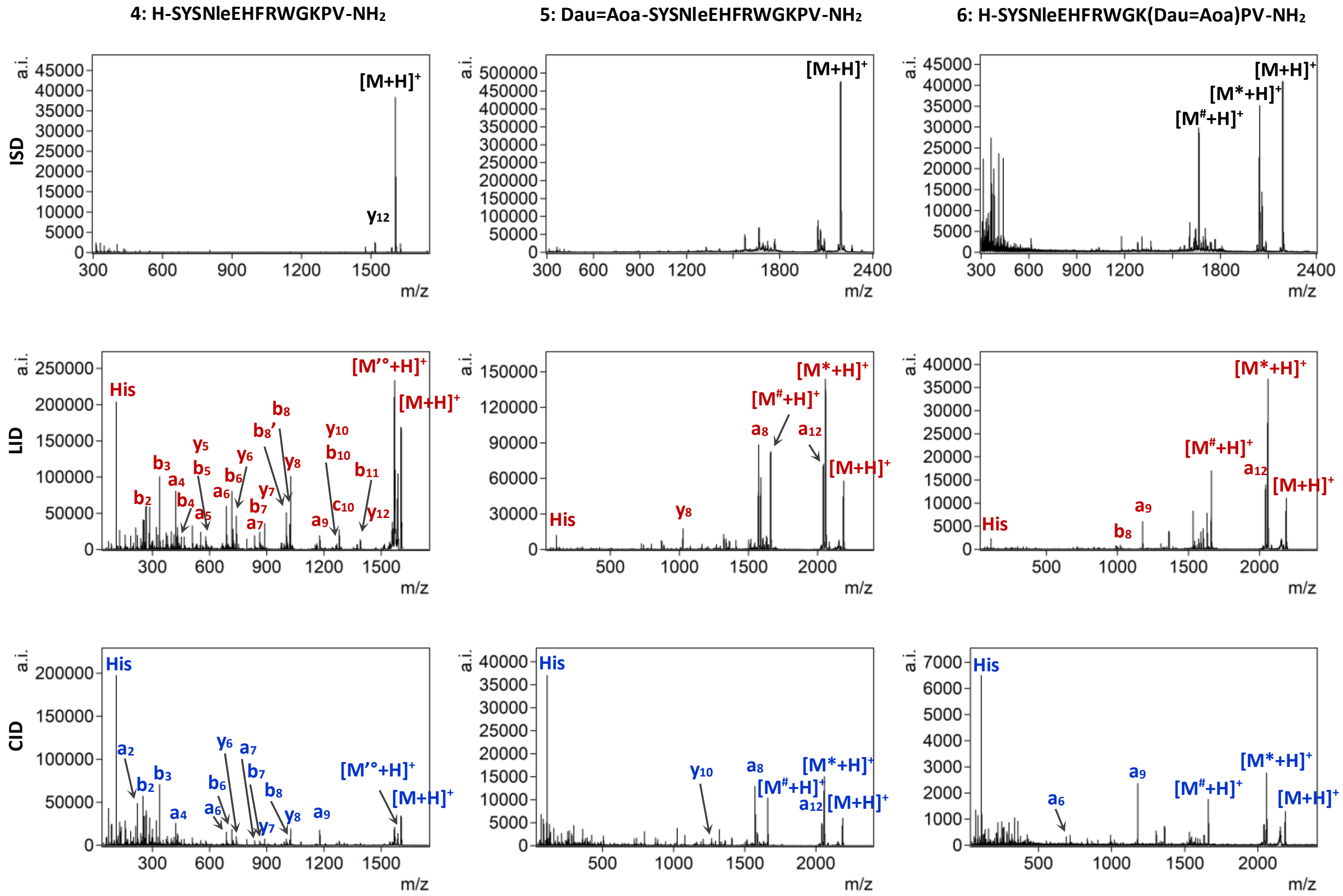

Figure S3: MALDI MS/MS spectra of the singly protonated compounds **4**, **5**, and **6**. A, B, C: MALDI-ISD spectra, D, E, F: MALDI-TOF/TOF LID spectra, G, H, I: MALDI-TOF/TOF CID spectra. Neutral losses are depicted as follows: *=sugar, ′=NH_3, °=H_2O, #=Dau+sugar, ¤=Dau+sugar+Aoa.

Table S1: Structural and analytical properties of the Angiopep-2 and Angiopep-2-bioconjugates.

	Compound	*m/z* calc. for $[M+3H]^{3+}$	*m/z* meas.[1] for $[M+3H]^{3+}$	Deviation (ppm)	R_t (min)[2]
**7**	H-TFFYGGSRGKRNNFKTEEY-OH	767.7064	767.7057	0.9	6.2
**8**	Dau=Aoa-TFFYGGSRGKRNNFKTEEY-OH	961.7681	961.7666	1.6	8.3
**9**	H-TFFYGGSRGK(Dau=Aoa)RNNFKTEEY-OH	961.7681	961.7665	1.7	7.6
**10**	H-TFFYGGSRGKRNNFK(Dau=Aoa)TEEY-OH	961.7681	961.7674	0.7	7.9

[1] ESI-MS data measured on a Thermo Scientific Q Exactive Focus mass spectrometer. [2] Analytical UPLC was performed using a Waters Acquity UPLC BEH C18 1.7 µm column (2.1 × 50 mm), at 40 °C; eluent A: water + 0.1% formic acid, eluent B: 80% acetonitrile + 0.1% formic acid. The following gradient was used: 0 min 2% B, 1 min 2% B, 17 min 100% B, 17.5 min 100% B, 18 min 2% B, 21 min 2% B. The flow rate was 300 µL min^{-1}.

MS spectra of the compounds 1-10:

Neutral losses are depicted as follows: *=sugar, °=H_2O.

1:

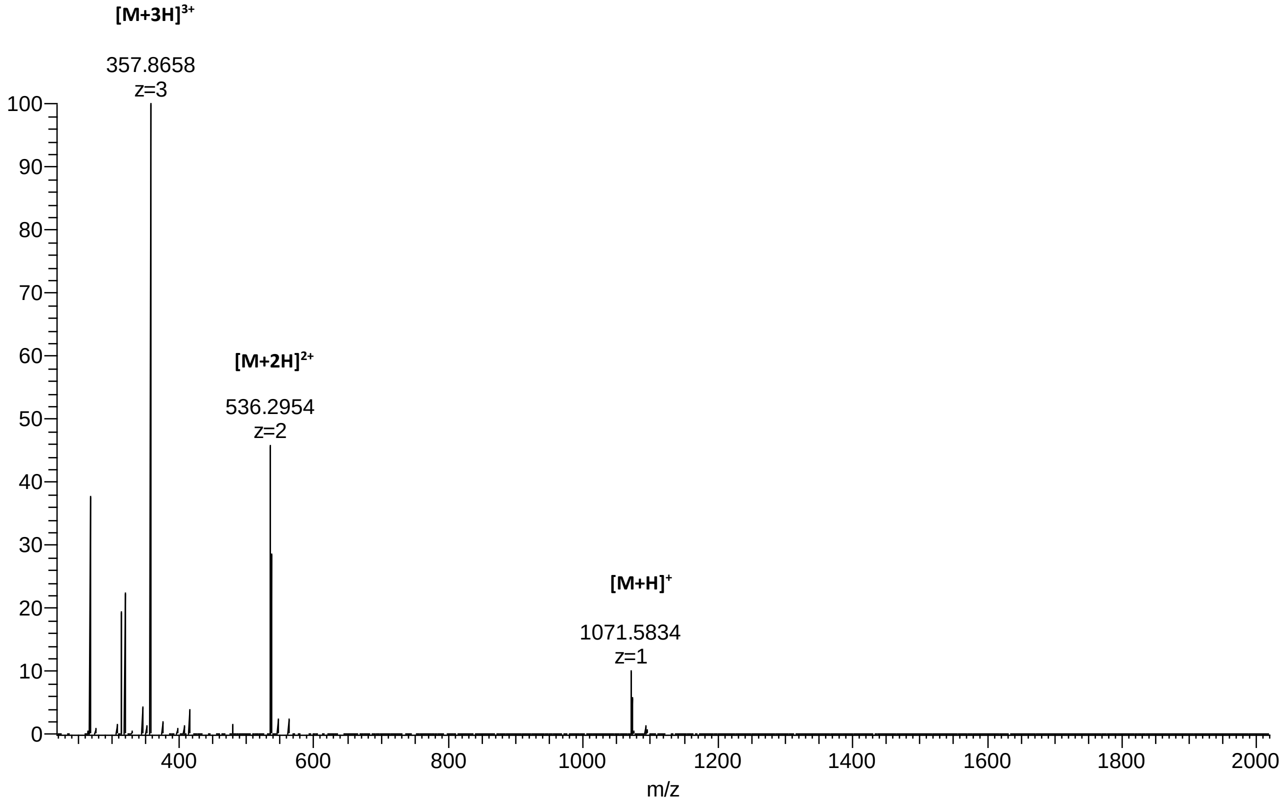

2:

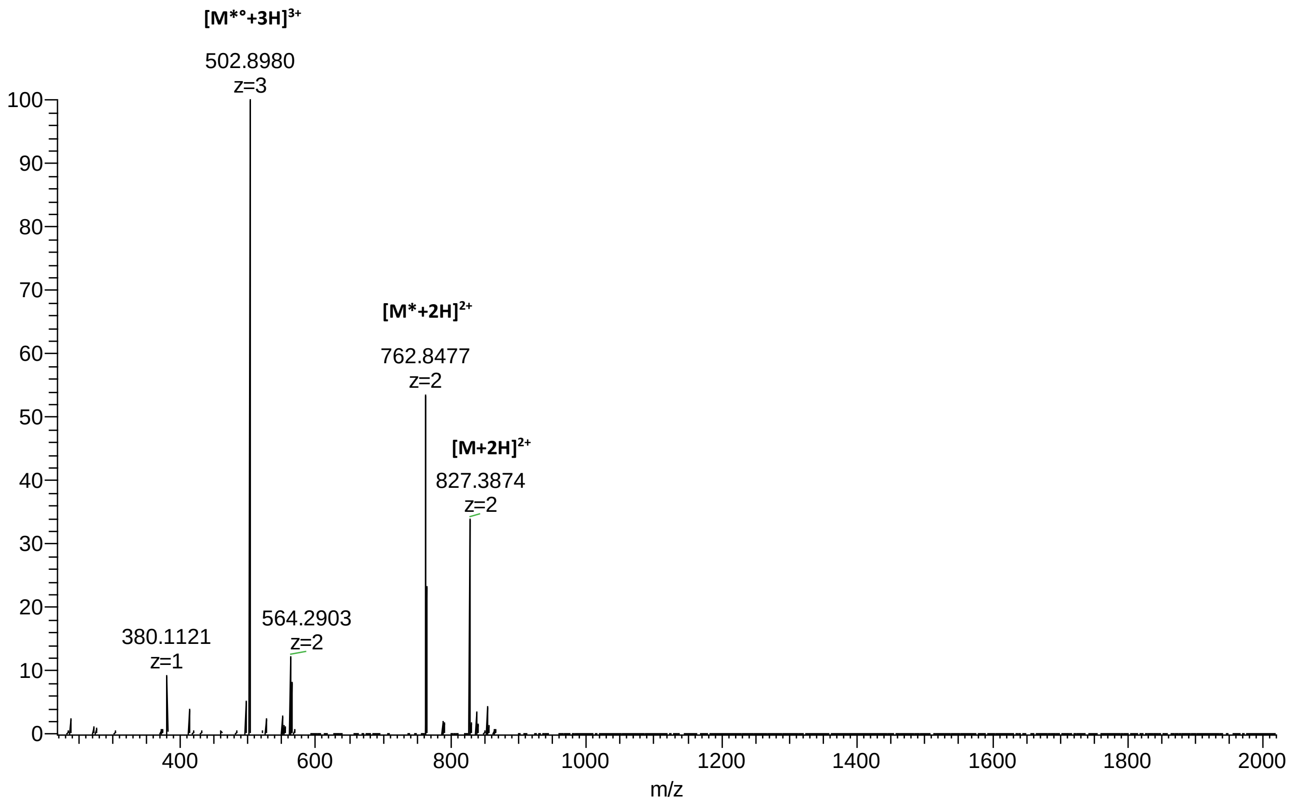

3:

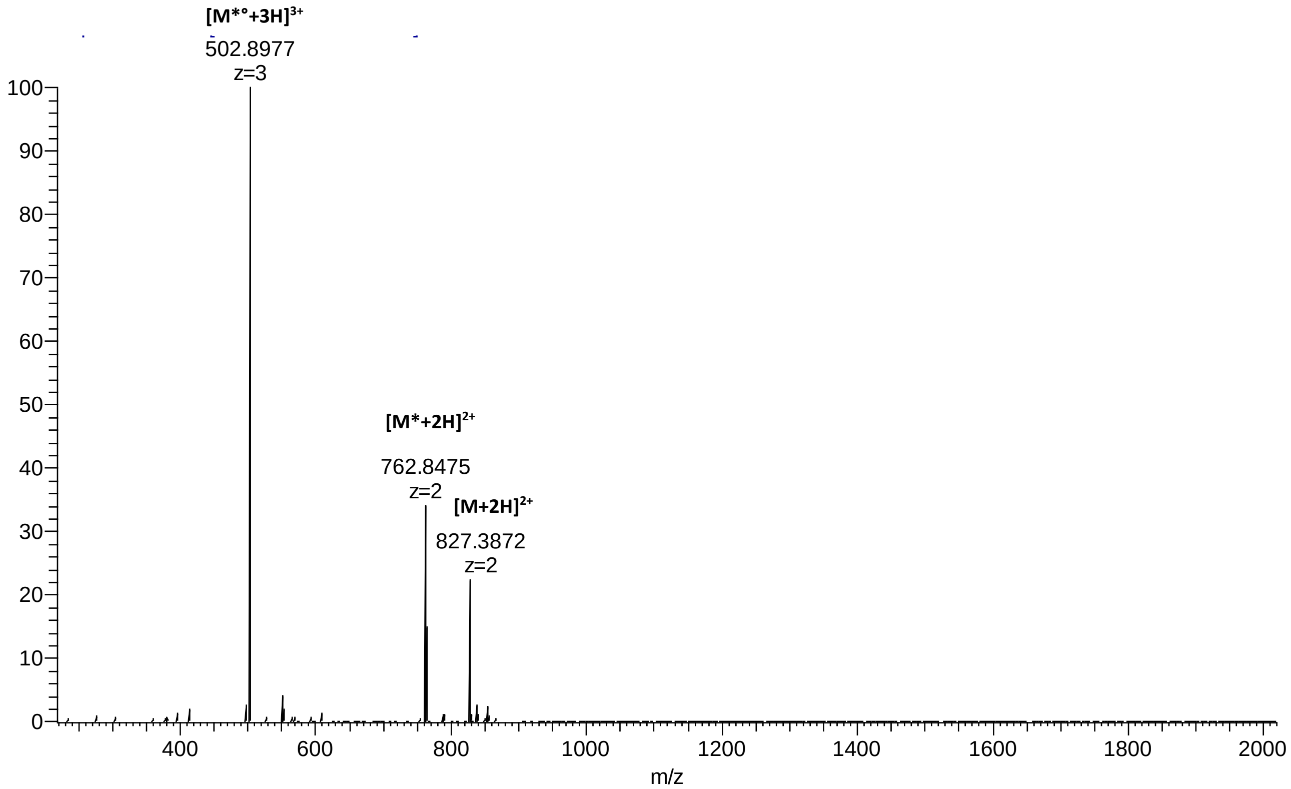
[M*°+3H]3+
502.8977
z=3
[M*+2H]2+
762.8475
z=2
[M+2H]2+
827.3872
z=2
m/z

4:

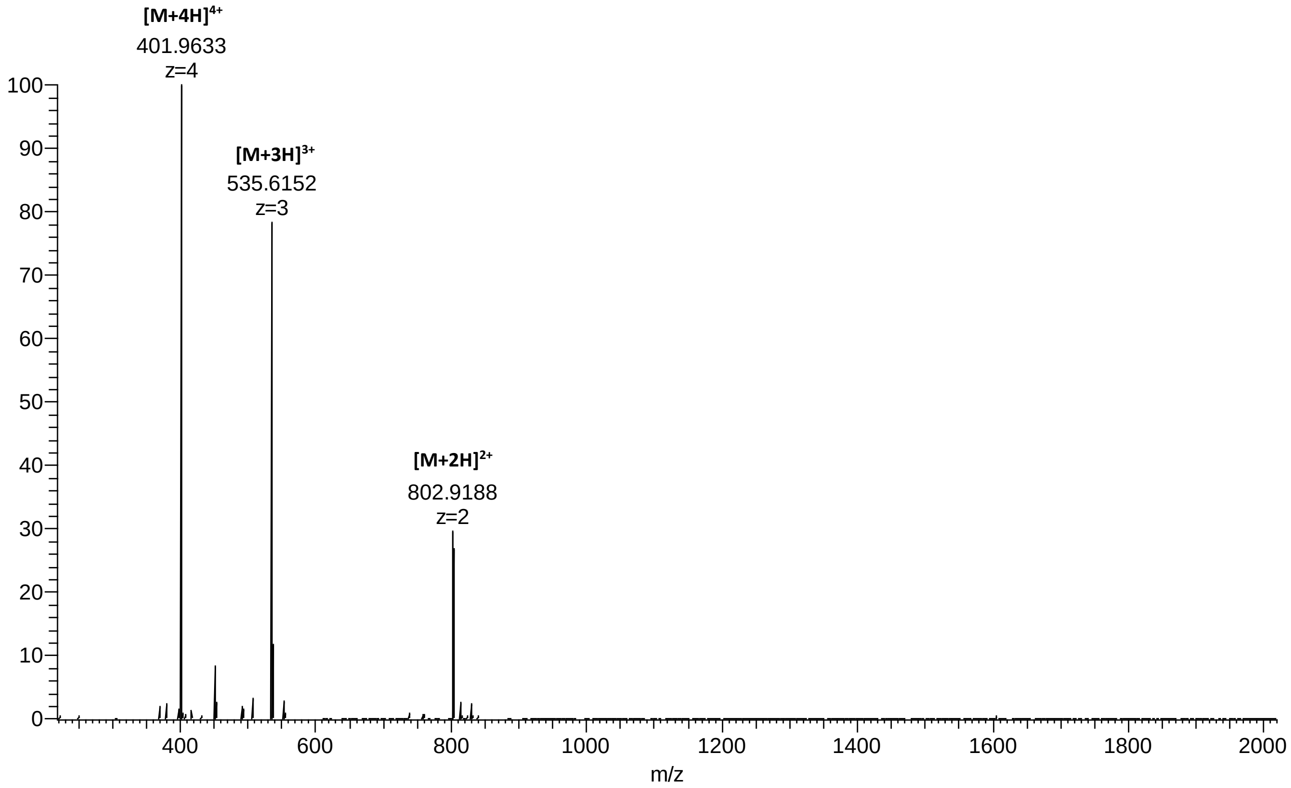
[M+4H]4+
401.9633
z=4
[M+3H]3+
535.6152
z=3
[M+2H]2+
802.9188
z=2
m/z

5:

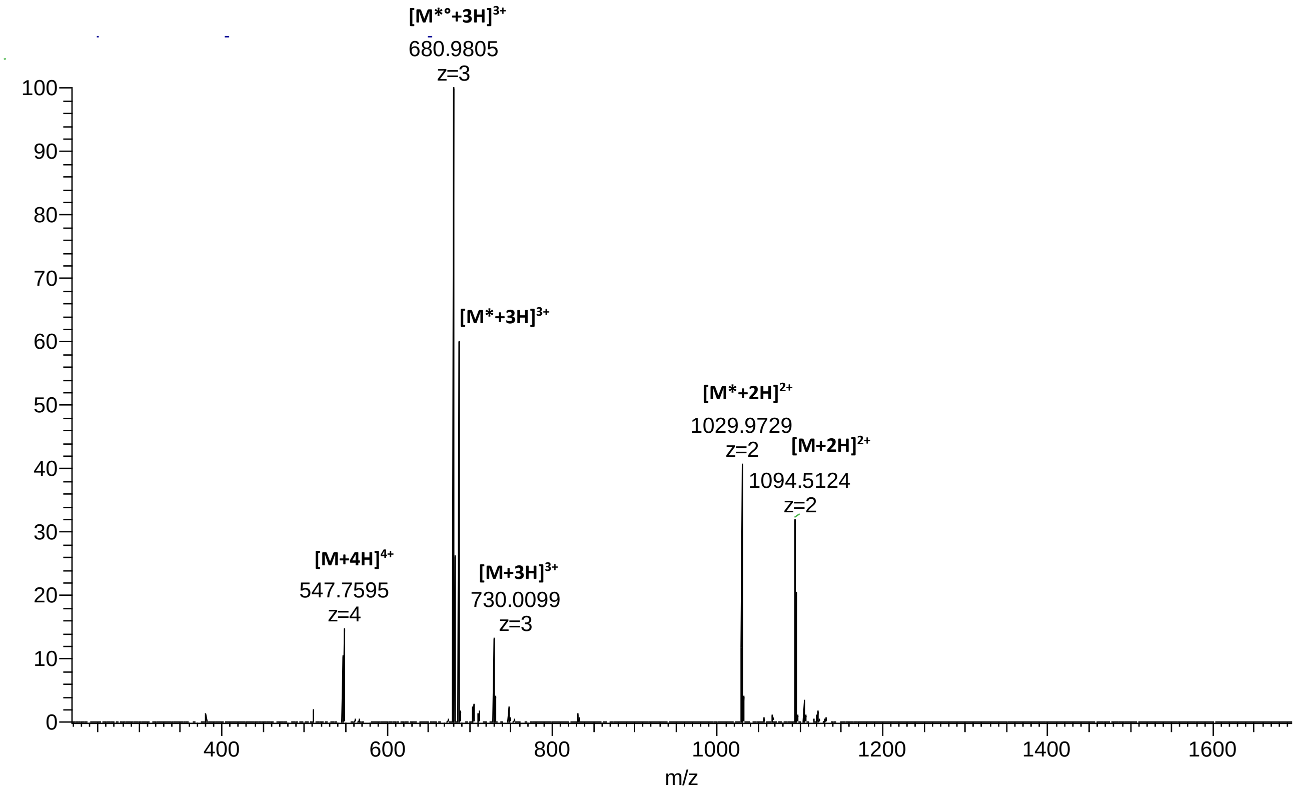
[M*°+3H]$^{3+}$
680.9805
z=3
[M*+3H]$^{3+}$
[M*+2H]$^{2+}$
1029.9729
z=2
[M+2H]$^{2+}$
1094.5124
z=2
[M+4H]$^{4+}$
547.7595
z=4
[M+3H]$^{3+}$
730.0099
z=3
m/z

6:

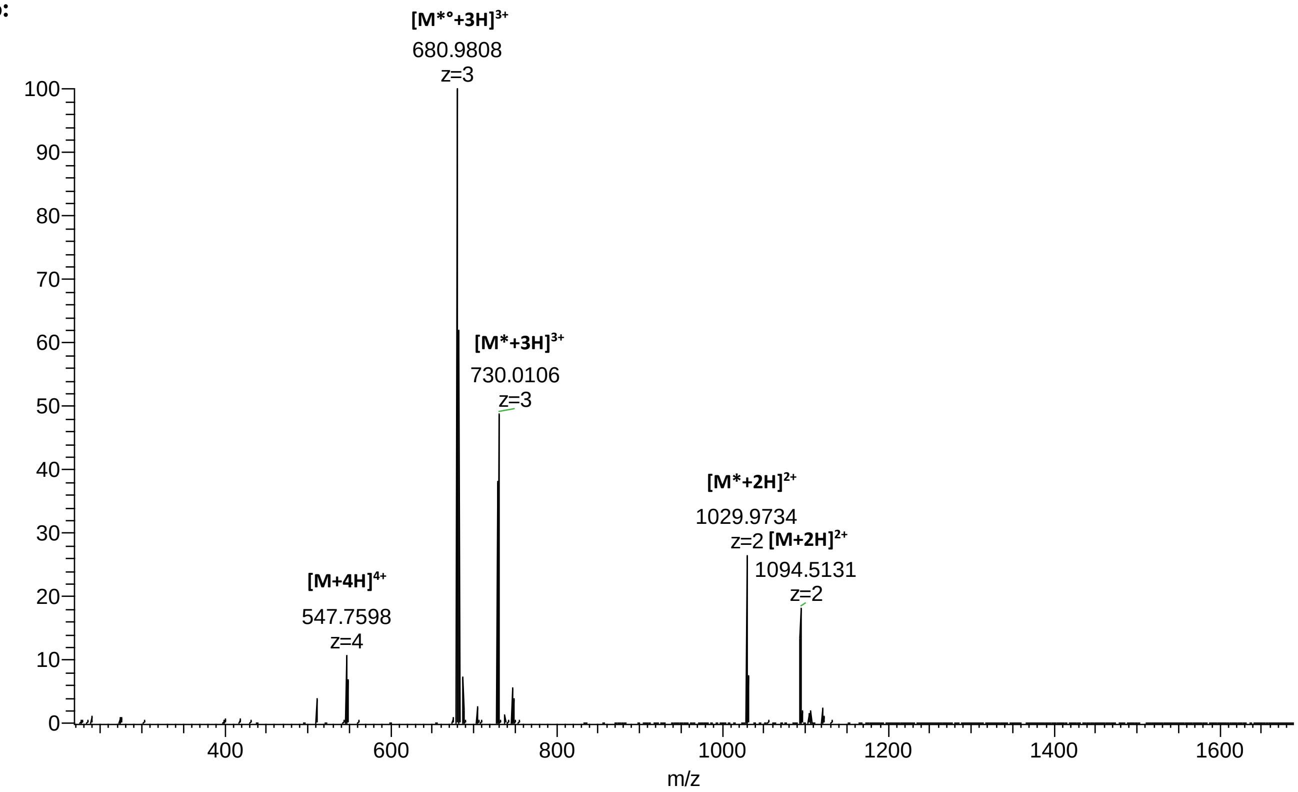
[M*°+3H]$^{3+}$
680.9808
z=3
[M*+3H]$^{3+}$
730.0106
z=3
[M*+2H]$^{2+}$
1029.9734
z=2
[M+2H]$^{2+}$
1094.5131
z=2
[M+4H]$^{4+}$
547.7598
z=4
m/z

7:

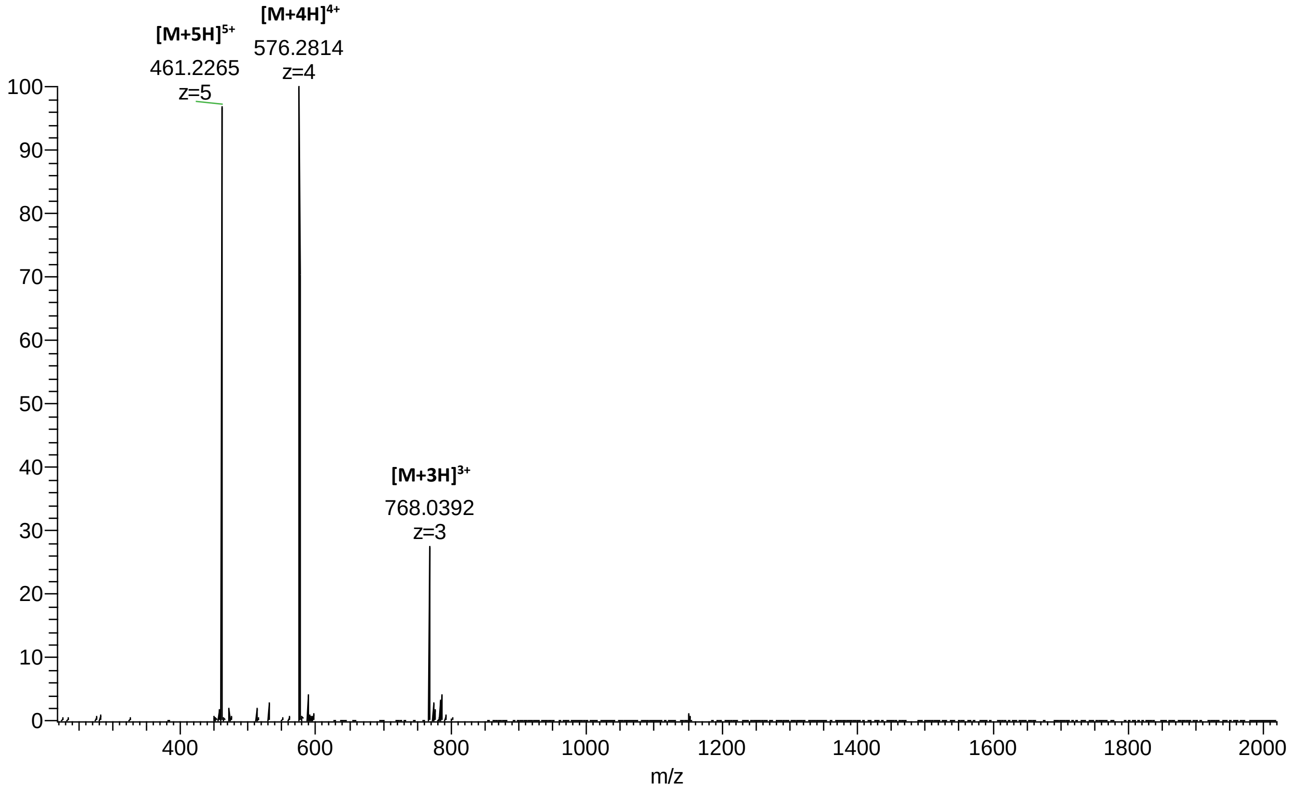
[M+5H]5+
461.2265
z=5
[M+4H]4+
576.2814
z=4
[M+3H]3+
768.0392
z=3
m/z

8:

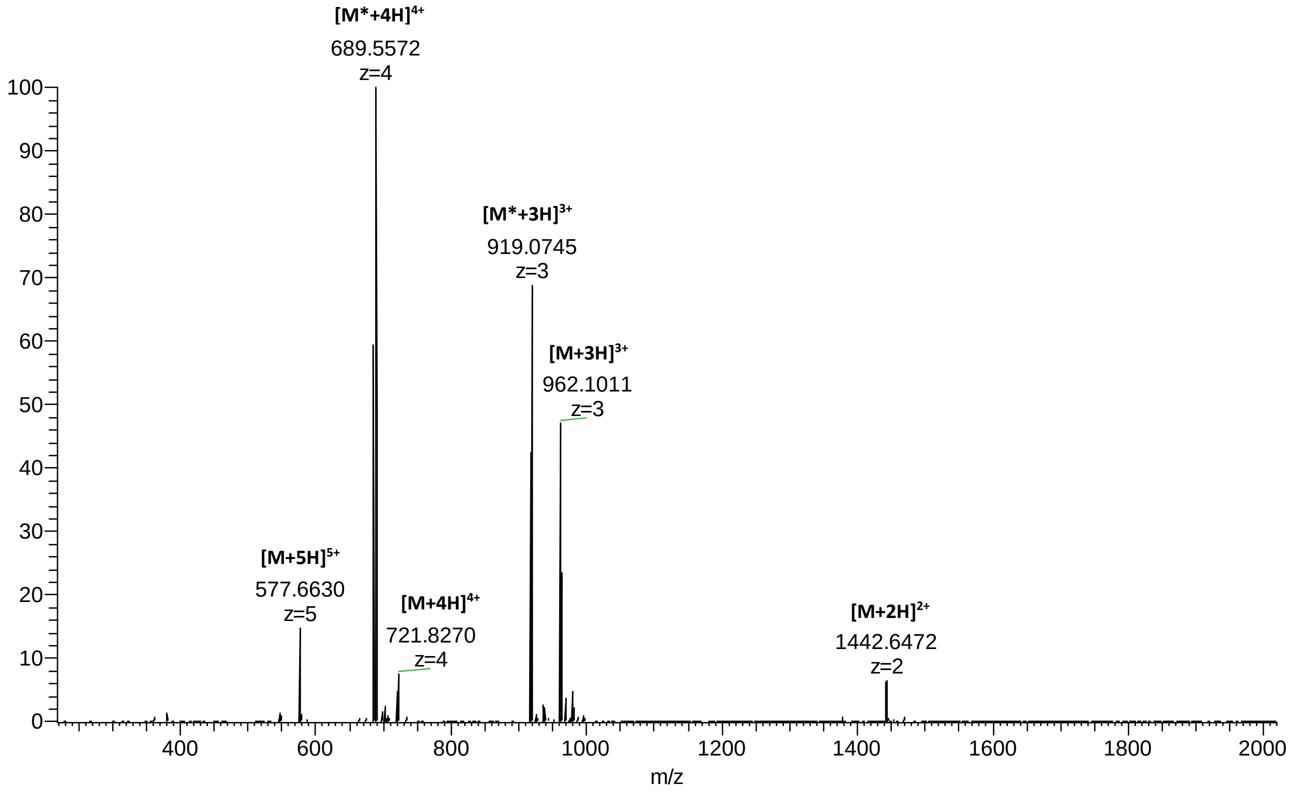
[M*+4H]4+
689.5572
z=4
[M*+3H]3+
919.0745
z=3
[M+3H]3+
962.1011
z=3
[M+5H]5+
577.6630
z=5
[M+4H]4+
721.8270
z=4
[M+2H]2+
1442.6472
z=2
m/z

9:

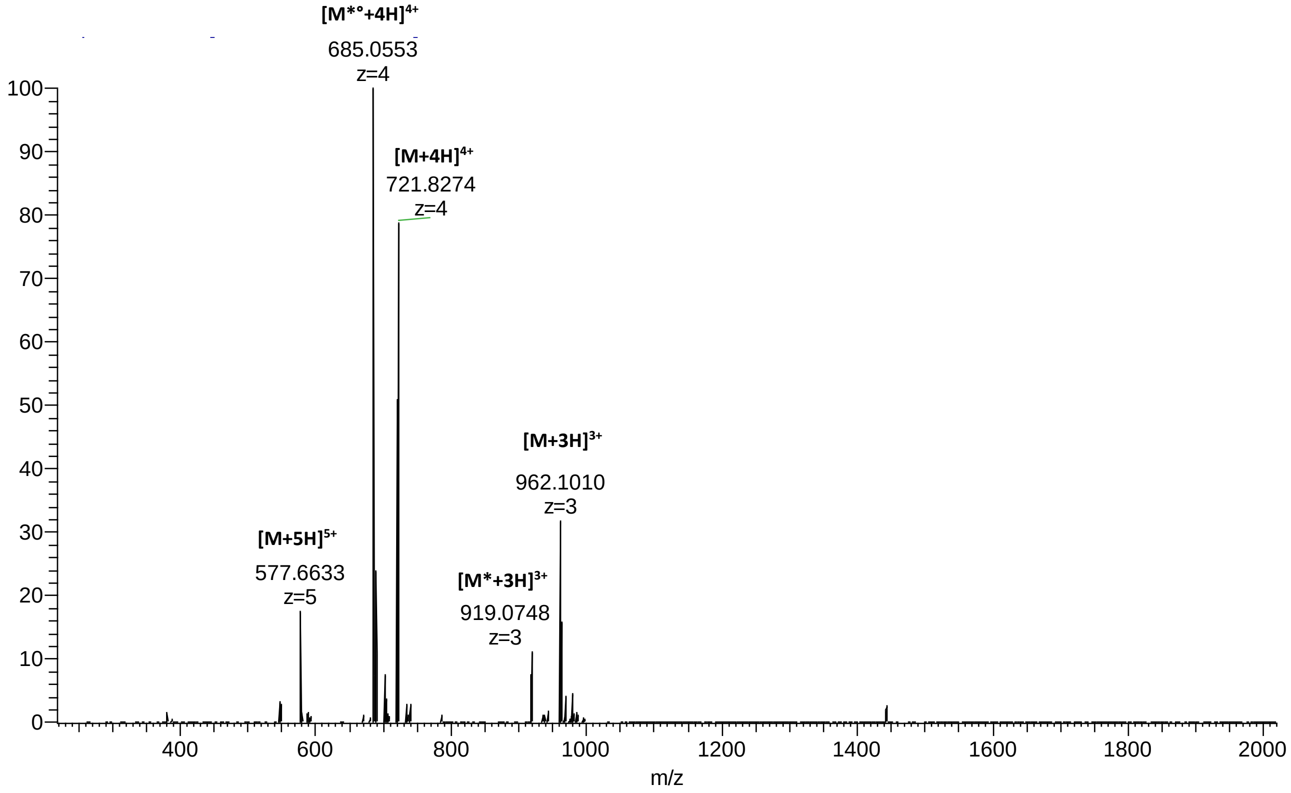
[M*°+4H]4+
685.0553
z=4
[M+4H]4+
721.8274
z=4
[M+3H]3+
962.1010
z=3
[M+5H]5+
577.6633
z=5
[M*+3H]3+
919.0748
z=3
m/z

10:

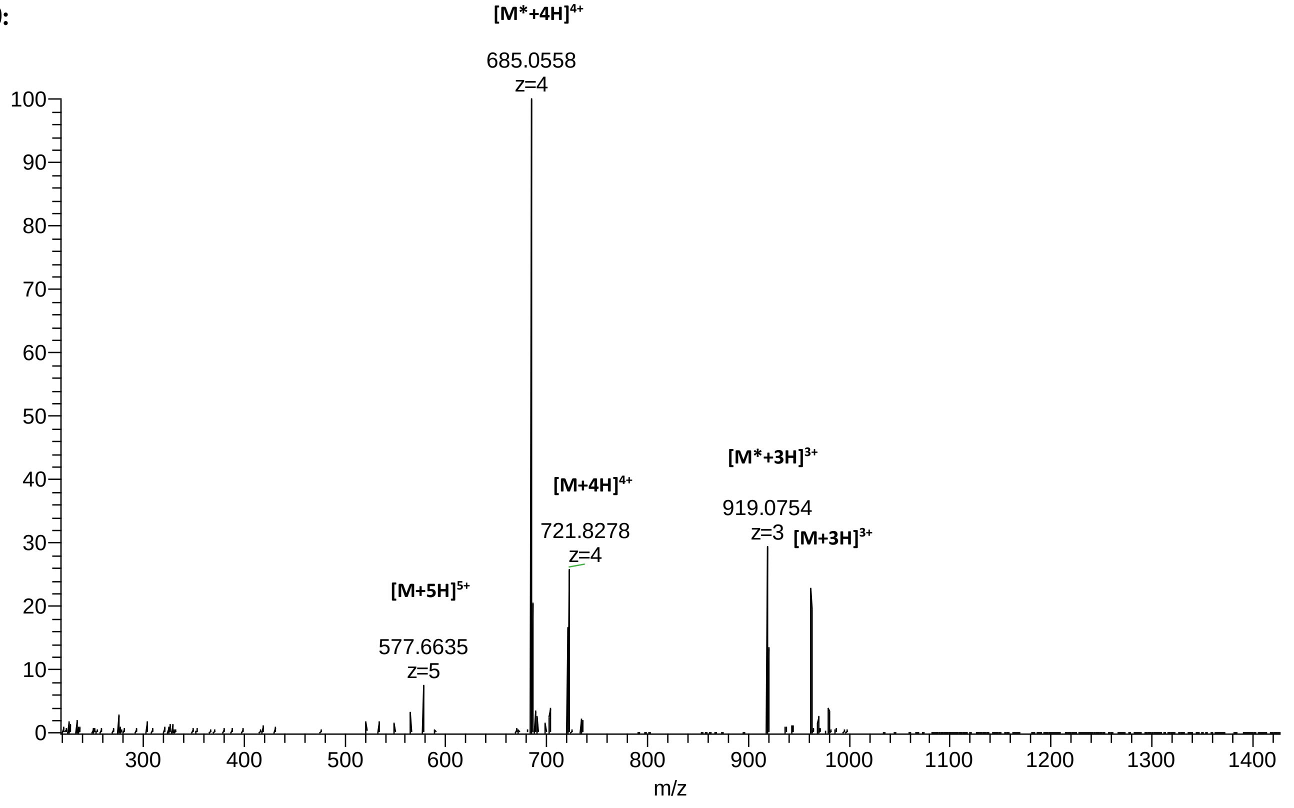
[M*+4H]4+
685.0558
z=4
[M*+3H]3+
919.0754
z=3
[M+4H]4+
721.8278
z=4
[M+3H]3+
[M+5H]5+
577.6635
z=5
m/z
